# A standardized stepwise approach to minimally invasive ileocolic anastomosis: Tips and tricks for laparoscopic and robotic surgery

**DOI:** 10.1111/codi.16159

**Published:** 2022-05-04

**Authors:** Alejandro Solís‐Peña, Arturo Cirera, Miquel Kraft Carré, Gianluca Pellino, Eloy Espín‐Basany

**Affiliations:** ^1^ Colorectal Surgery Vall d'Hebron University Hospital Universitat Autònoma de Barcelona (UAB) Barcelona Spain; ^2^ Department of Advanced Medical and Surgical Sciences Università degli Studi della Campania “Luigi Vanvitelli” Naples Italy

**Keywords:** ileocolic anastomosis, intracorporeal anastomosis, laparoscopic approach, robotic approach

## Abstract

**Aim:**

Intracorporeal anastomosis has been associated with earlier recovery of postoperative bowel function, shorter length of stay and lower surgical site infection rates. The aim of this work is to describe a step‐by‐step standardized technique for intracorporeal ileocolic and ileosigmoid anastomosis suitable for laparoscopic and robotic colectomy.

**Method:**

Each step of the technique is illustrated using a composite collection of three operative patient videos. Two procedures were performed robotically and one was laparoscopic. Tips are provided to construct a two‐layer anastomosis (both posteriorly and anteriorly). The procedures are presented in stepwise fashion, discussing the advantages and feasibility of the technique.

**Results:**

The standardized technique described herein was used in three patients for this report, of whom two underwent right colectomy and one subtotal colectomy for cancer. The median operating time was 255 (206–333) min. There were no intraoperative complications. No major postoperative complications or 30‐day readmissions occurred. The median length of stay was 4 (3–5) days.

**Conclusion:**

The described technique of a two‐layer anastomosis can be used with any available minimally invasive approach. It is safe and feasible. Using a standardized approach, the technique can be easily taught and mastered, optimizing operating times and reducing adverse events.

## INTRODUCTION

Several studies have demonstrated the advantages of minimally invasive surgery compared with open surgery after an oncological colorectal resection [e.g. fewer surgical site infections (SSI), less use of analgesics, etc.] with equivalent results. By means of these findings, the minimally invasive approach is now considered by many groups to be the standard of care for colorectal cancer [1]. Both laparoscopic right colectomy and extended right colectomy are well‐established procedures for the treatment of right‐sided colon neoplasms [2]. As with right colectomy, the laparoscopic approach shows the same advantages in subtotal colectomy [3]. Robotic‐assisted procedures seem to have reduced conversion rates in ‘difficult cases’ and facilitate the procedure by means of increased dexterity and range of motions [4]. Ileocolic anastomoses can be performed using several techniques, for example extracorporeal versus intracorporeal, mechanical versus hand‐sewn and single‐ versus double‐layered. Intracorporeal anastomosis has potential advantages over extracorporeal, such as reduced traction on the mesentery, full visualization of every step with the reduction of ileum twist or specimen extraction via smaller incisions, with better cosmesis [2, 5, 6]. Although there are many ways to fashion an intracorporeal anastomosis, some studies have demonstrated that a double‐layered anastomosis reduces the clinical impact of anastomotic leaks [7]. Both laparoscopic and robotic intracorporeal anastomosis have been associated with earlier return of bowel function, shorter length of stay, lower morbidity and lower SSI rates [5, 8]. Thus, the main indications for this technique are ileocolic and ileosigmoid anastomosis after right, extended right and subtotal colectomies, respectively.

## METHOD

We present our standardized technique of intracorporeal, stapled, ileocolic and ileosigmoid anastomosis reinforced on both sides with a hand‐sewn suture, thereby creating a ‘double‐layered’ (both anteriorly and posteriorly) anastomosis. Video S1 (parts 1 and 2) shows a step‐by‐step technique for performing the anastomosis both with laparoscopy and robotic‐assisted surgery. At our hospital we use the DaVinci Xi™ platform (Intuitive Surgical™). Each step is illustrated using a composite collection of three operative patient videos. Two procedures were performed robotically and one was laparoscopic.

### Patient positioning

After the entire dissection has been completed and both the ileum and colon have been sectioned either robotically or laparoscopically, with the patient in a supine position, the lateral tilt is removed (both during right colectomy and subtotal colectomy). During laparoscopic surgery, the operating surgeon stands on the left of the patient, with the assistant surgeon on his/her left side; during robotic surgery, the assistant is on the left of the patient while the operating surgeon sits at the console.

### Port placement

**FIGURE 1:**
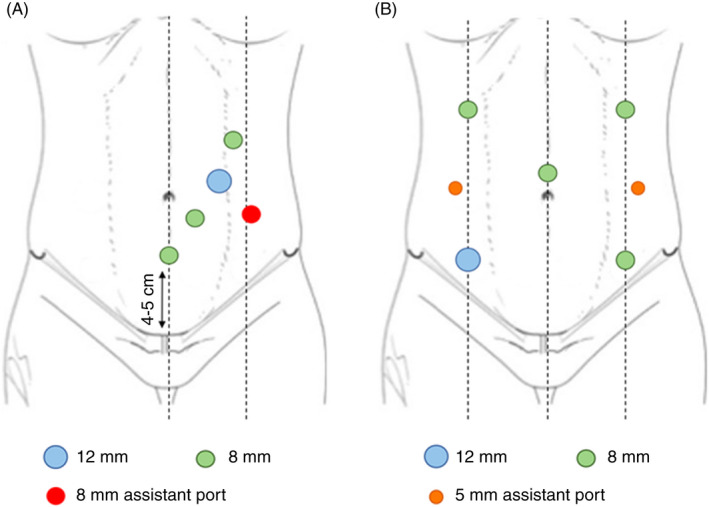
Trocar placement for robot‐assisted right colectomy (A) and for robot‐assisted subtotal colectomy (B)

In robotic‐assisted right colectomy five trocars are positioned as follows: one 8 mm port is placed in the midline, 4–5 cm above the pubis; then, three robotic trocars are placed cranially between the midline and midclavicular line (two 8 mm ports and a 12 mm port in position 3 or 4). In addition, one 8 mm laparoscopic assistant trocar is placed at the level of the midclavicular left line, between trocars 2 and 3 (Figure 1).

A total of seven trocars are required to perform fully robotic subtotal colectomy. These comprise four 8 mm robotic ports located respectively in both upper quadrants, the lower left quadrant and periumbilical. The 12 mm fifth robotic trocar is located, as shown in the figure, in the right lower quadrant. In addition, two 5 mm laparoscopic assistance trocars are used, located in both flanks (Figure 1B).

**FIGURE 2:**
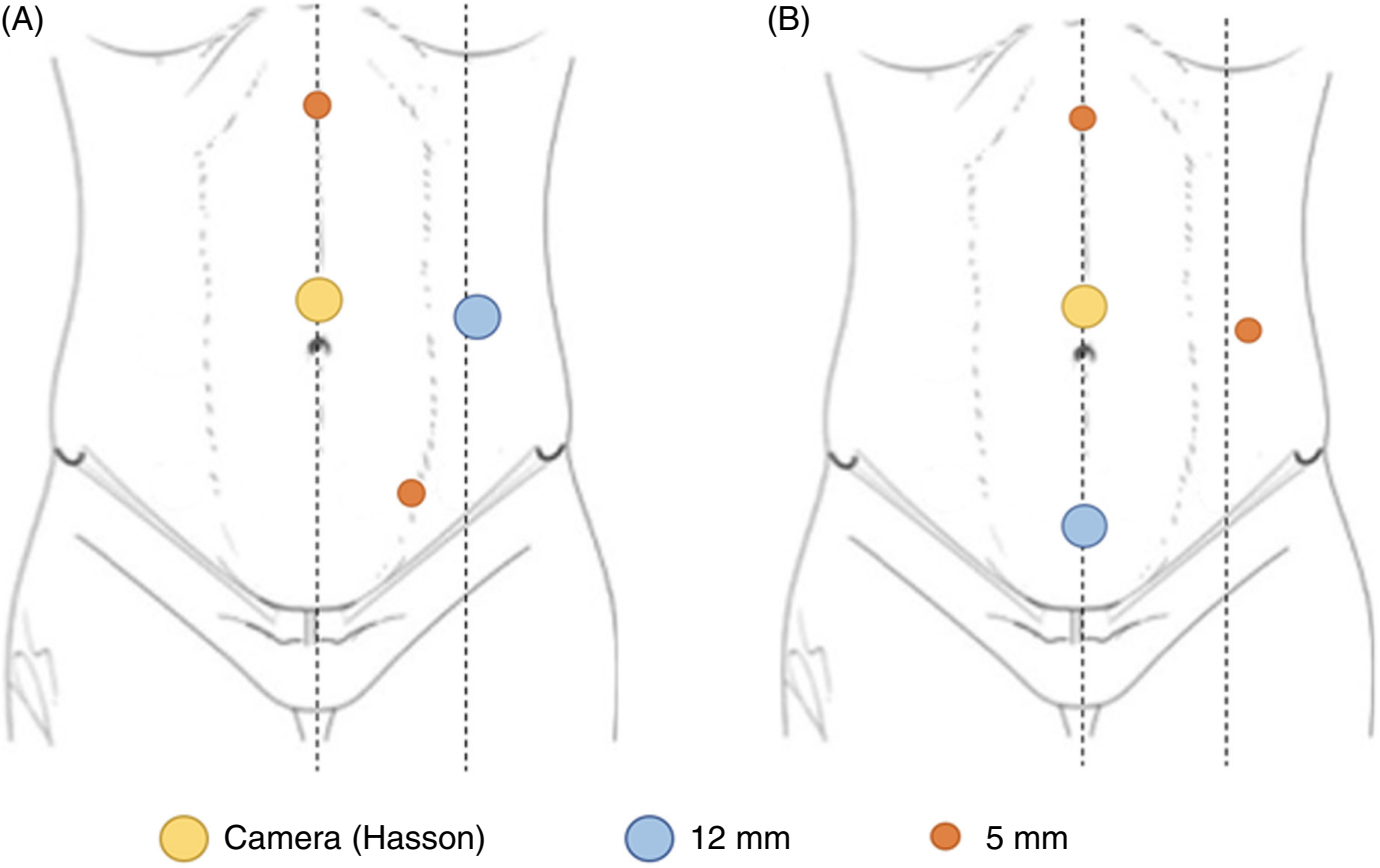
Trocar placement for laparoscopic right colectomy (A, standard technique; B, alternative disposition)

In the laparoscopic technique, two different alternatives for trocar placement are suggested. In the first one, a periumbilical Hasson port is placed, through which the camera is inserted. Next, a 12 mm trocar is positioned in the left flank and two 5 mm ports go in the epigastrium and the left lower quadrant, respectively (Figure 2A). Alternatively, the 12 mm trocar can be positioned just above the pubis, so that the 5 mm port would move towards the left flank (Figure 2B).

### Docking

The robot is at the right side of the patient at a 90° angle during the right colectomy, and between the legs during subtotal colectomy. The robot arms are aligned with the trocars and docking is performed. Robotic instruments are introduced under direct vision.

### Surgical steps

The anastomosis is started with a running suture of the posterior plane using a barbed thread. Once the posterior component of the anastomosis has been completed, an ileal and a colonic enterotomy are performed with monopolar energy. A side‐to‐side mechanical anastomosis is fashioned using an Endostapler. Another continuous barbed suture is used to close the defect, starting from the closest corner to the posterior suture and ending a few centimetres beyond the end of the buttonhole. The standardized technique also includes an anterior reinforcement using a continuous barbed suture.

## COMPARISON WITH OTHER METHODS, ADVANTAGES AND DISADVANTAGES

Compared with other ileocolic anastomotic techniques, our procedure has been standardized to be utilized by all surgeons in our team, including surgeons in training and fellows. Intra‐ and interoperator variabilities are eliminated, likely reducing postoperative complications related to the anastomosis. The adoption of a homogeneous approach facilitates the learning process for surgical trainees and audit of the skills training process.

The use of the ‘double layer’ or hand‐sewn reinforcement of the anterior and posterior sides of the anastomosis has not been associated with reduced rates of anastomotic leak, but it has reduced the need for reoperation in the case of anastomotic leak, that is, it reduces the clinical impact of an anastomotic leak [7].

With the current technique, the closure of the enterotomy is achieved with a double‐layer, full‐thickness, running barbed suture. Some studies have demonstrated the feasibility and safety of this type of suture, with similar complication rates of conventional suture closure [9, 10]. In addition, in a multicentre case–control study published in 2020 with 1092 patients who underwent right colectomy with ileocolic anastomosis, a significant reduction was found on both anastomotic leak and bleeding with the running suture performed with a barbed suture thread. No differences were seen between laparoscopic and the robotic‐assisted approaches [11].

Lastly, intracorporeal anastomosis, compared with the extracorporeal approach, not only has similar rates of anastomotic leak but has also been shown to lower the risk for global, medical and surgical complications, SSI and wound complications, including the rate of incisional hernias, and to shorten the hospital stay [2, 12, 13, 14].

## RESULTS

We describe data from three different patients diagnosed with nonmetastatic colorectal cancer and who were operated on either by laparoscopy or robotic‐assisted surgery: two cases of right colectomy and one case of subtotal colectomy. After the colonic resection, an ileocolic anastomosis following the standardized technique was performed in all cases. Pathology was consistent with colon adenocarcinomas in all patients. In all cases, a Pfannenstiel incision was performed for the specimen extraction because it has a lower incidence of incisional hernia as well as better cosmetic results. Demographic and operative details of the patients are listed in Table 1. No major intra‐ and postoperative complications were observed, including ones related to the anastomotic status (leak, bleeding or stricture). The median length of hospital stay was 4 days. Detailed surgical and postoperative outcomes are shown in Table 1.

**TABLE 1:** Patient characteristics, surgical details, and postoperative outcomes

	Clinical case 1	Clinical case 2	Clinical case 3
Age (years)	74	38	68
Gender	Female	Male	Male
Body mass index (kg/m^2^)	28.3	26.9	27.7
Location of the tumour	Right colon	Right colon	Mid‐transverse colon
Signs and symptoms at diagnosis	Asymptomatic	Microcytic anaemia	Asymptomatic
Surgical details			
Type of surgery	Robot‐assisted right colectomy	D3 laparoscopic right colectomy	Robot‐assisted subtotal colectomy
Duration of surgery (min)	206	255	333
Blood loss (ml)	<100	<100	<100
Intraoperative complications	None	None	None
Outcomes			
Postoperative complications	None	None	Nausea and vomiting
Hospital stay (days)	3	4	5
Pathology	Adenocarcinoma (pT3 pN1a pM0)	Adenocarcinoma (pT3 pN0 pM0)	Adenocarcinoma (pT3 pN0 pM0)

## CONCLUSIONS

The minimally invasive intracorporeal ileocolic anastomosis technique is an effective, feasible and safe procedure. The described technique makes it replicable regardless of the approach used (laparoscopic or robotic) and harmonizes a complex procedure, being particularly suitable for training units or academic hospitals.

## CONFLICT OF INTEREST

The authors declare that they have no conflict of interests.

## AUTHOR CONTRIBUTIONS

ASP, AC, MKC collected data, prepared the video, and drafted the manuscript. GP and EEB critically revised the article. All authors approved the last version that was accepted for publication.

## ETHICAL APPROVAL

This work adhered to the Declaration of Helsinki. All patients provided consent before undergoing the surgical procedure.

## Supporting information


Video S1

## Data Availability

The data that support the findings of this study are available from the corresponding author upon reasonable request.
